# CCAAT/enhancer binding protein delta (C/EBPδ) deficiency does not affect bleomycin-induced pulmonary fibrosis

**Published:** 2018-02-21

**Authors:** Duitman JanWillem, Cong Lin, Sophie Moog, Madeleine Jaillet, Yves Castier, Aurélie Cazes, Keren S. Borensztajn, Bruno Crestani, C. Arnold Spek

**Affiliations:** *1 INSERM UMR1152, Medical School Xavier Bichat, Paris, France*; *2 Université Paris Diderot, Sorbonne Paris Cité, Département Hospitalo-universitaire FIRE (Fibrosis, Inflammation and Remodeling) and LabEx Inflamex, Paris, France*; *3 Center for Experimental and Molecular Medicine, Academic Medical Center, Amsterdam, the Netherlands*; *4 Assistance Publique-Hôpitaux de Paris (APHP), Hôpital Bichat, Service de Pneumologie A, Paris, France*; *5 INSERM UMR _S933, Université Pierre et Marie Curie, Paris, France*

**Keywords:** Pulmonary fibrosis, IPF, transcription factor, C/EBPδ.

## Abstract

**Background:**

Idiopathic pulmonary fibrosis is a devastating fibrotic diffuse parenchymal lung disorder that remains refractory to pharmacological therapies. Therefore, novel treatments are urgently required. CCAAT/enhancer binding protein delta (C/EBPδ) is a transcription factor that mediates critical cellular functions in pathophysiology and which was recently suggested to be a key regulatory component in IPF. The purpose of this study was to prove or refute the importance of C/EBPδ in pulmonary fibrosis.

**Methods:**

Pulmonary fibrosis was induced by intranasal instillation of bleomycin into wild-type and C/EBPδ deficient mice. At different time intervals after bleomycin instillation, fibrosis was assessed by hydroxyproline analysis, histochemistry and q-PCR for fibrotic marker expression.

**Results:**

C/EBPδ deficient mice developed pulmonary fibrosis to a similar degree as wildtype mice as evident from similar Ashcroft scores, hydroxyproline levels and expression levels of collagen, fibronectin and α-smooth muscle actin at both 14 and 21 days after bleomycin instillation. The resolution of fibrosis, assessed at 48 days after bleomycin instillation, was also similar in wildtype and C/EBPδ deficient mice. In line with the lack of effect of C/EBPδ on fibrosis progression/resolution, macrophage recruitment and/or differentiation were also not different in wildtype or C/EBPδ deficient mice.

**Conclusions:**

Overall, C/EBPδ does not seem to affect bleomycin-induced experimental pulmonary fibrosis and we challenge the importance of C/EBPδ in pulmonary fibrosis.

**Relevance for patients:**

This study shows that the transcription factor C/EBPδ does not play a major role in the development of pulmonary fibrosis. Pharmacological targeting of C/EBPδ is therefore not likely to have a beneficial effect for patients suffering from pulmonary fibrosis.

## 1. Introduction

Idiopathic pulmonary fibrosis (IPF), the most common form of pulmonary fibrosis, is a progressive and fatal disease that is characterized by excessive extracellular matrix (ECM) production [1-3]. The prevalence of IPF ranges from 14-42 cases per 100,000 persons depending on the criteria used for diagnosis. IPF patients have a dismal prognosis with a median survival of 2 to 3 years and a 5 year mortality rate of 70-80% that exceeds many types of cancer. Treatment modalities for IPF are limited and lung transplantation is the last resort, which is however for selected patients only. Recently, two novel drugs, i.e. pirfenidone and nintedanib, which both significantly reduce the decline of lung function in patients with mild to moderate IPF were introduced into the clinic [4,5]. However, both drugs have serious side effects and do not stop nor reverse the disease. Novel treatment options are thus eagerly awaited [6].

CCAAT-enhancer-binding protein delta (C/EBPδ), also known as nuclear factor interleukin (IL)-6β, is a member of the C/EBP family of transcription factors containing six members, C/EBPα, C/EBPβ, C/EBPδ, C/EBPγ, C/EBPε and C/EBPζ [7]. All these members, except C/EBPζ, consist of an N-terminal transactivation domain, a basic DNA binding domain and a C-terminal leucine zipper domain that allows homo- or hetero-dimerization of the different members. Originally identified as a transcription factor that is rapidly upregulated during the acute phase response [8], C/EBPδ is now well established to act as a pleiotropic transcription factor involved in many biological processes like, amongst others, cellular differentiation, proliferation and inflammation [9]. Considering the importance of these key cellular processes in pulmonary fibrosis, it is tempting to speculate that C/EBPδ would modify disease progression in IPF and a recent integrated genomic analysis confirmed this notion by identifying C/EBPδ as a key regulatory component in IPF [10].

In line with a potential important role of C/EBPδ in pulmonary fibrosis, C/EBPδ recently emerged as a key player in macrophages. Indeed, C/EBPδ potentiates macrophage recruitment during *Klebsiella* -induced pulmonary infection [11], it modulates cytokine expression in macrophages [12,13] and knockdown of C/EBPδ expression diminishes M1 macrophage activation whereas it enhances M2 macrophage polarization [14]. The role of C/EBPδ in macrophage biology may be particularly relevant as macrophages are known to be key regulators in the progression of pulmonary fibrosis [15-19]. Indeed, macrophage recruitment is an early event following lung injury and M2 macrophages secrete large amounts of profibrotic cytokines like TGF-β and PDGF [20]. These profibrotic cytokines on its turn induce fibroblast proliferation and differentiation into collagen-secreting myofibroblasts ultimately leading to ECM deposition and fibrosis [19].

Overall, C/EBPδ thus seems to be a key regulator of cellular processes involved in pulmonary fibrosis. In the current manuscript, we consequently explored the role of C/ EBPδ in experimental pulmonary fibrosis. Surprisingly, we show that C/EBPδ deficiency has no effect on bleomycin-induced fibrosis and we thus challenge the importance of C/ EBPδ in pulmonary fibrosis.

## 2 Materials and Methods

*Human mRNA samples* - Lung tissue was obtained from 38 patients with IPF (4 women, 34 men; mean age, 61.0 ± 7.6 yr), and 28 control subjects (patients undergoing lung surgery for removal of a primary lung tumor; 8 women, 20 men, mean age 62.8 ± 12.8 yr). Control tissues were obtained from a noninvolved segment, remote from the solitary tumor lesion, and normalcy of these control lungs was verified histologically as described previously [21]. Patient demographics are listed in table 1. This study was approved by the local ethics committee (CCP Ile de France 1, no. 0811760. Written informed consent was obtained from all subjects. Total RNA was isolated using a Nucleospin RNA isolation kit (Macherey-Nagel, Düren, Germany) according the manufacturer’s recommendations.

**Table 1.** Patient demographics of samples included in the study. FVC: forced vital capacity, DLCO: diffusing capacity of the lung for carbon monoxide (CO), Stdev: standard deviation. 1For 5 control and 4 IPF patients FVC data were not available. 2For 9 control and 9 IPF patients DLCO data were not available. 3For 5 control and 1 IPF patient(s) smoking history was not available.

Human sample demographics	Control (n = 28)	IPF (n = 38)
Gender (males/females)	20/8	34/4
Age (years; mean ?Stdev)	62.8 ?12.8 yr	61.0 ?7.6 yr
FVC (% of predicted; mean ?Stdev)1	96.2 ?17.0	56.5 ?16.6
DLCO (% of predicted; mean ?Stdev %)2	80 ?19.3	31.0 ?15.3
Smoking history (yes/no)3	21/2	31/6

*Animal model of pulmonary fibrosis* - Specific pathogen-free 8- to 12 week old C57BL/6 mice were purchased from Charles River and C/EBPδ deficient mice (on a C57BL/6 background; generated as described previously [22]) were bred in the animal facility of the Academic Medical Center with free access to food and water. Mice used for experiments were age and sex matched. The Animal Care and Use Committee of the University of Amsterdam approved all animal experiments. Pulmonary fibrosis was induced using bleomycin essentially as described before [23,24] and mice were sacrificed 14, 21 or 48 days after bleomycin instillation.

Hydroxyproline Assay - Hydroxyproline analysis was performed by the hydroxyproline assay kit as per the manufacturer’s instructions (Sigma, Netherlands) and as described before [23].

*Histological Analysis* - Histological examination was performed essentially as described before [25]. Briefly, the excised lung was fixed in formalin, embedded in paraffin and 4-μm-thick slides were subsequently deparaffinized, rehydrated and washed in deionized water. Slides were stained with hematoxylin and eosin (H&E) according to routine procedures after which the severity of fibrosis was assessed according to the Ashcroft scoring system [25] using a 100× magnification. Two independent observers were blinded to the treatment group and an average of 10 fields of each lung section was selected and scored. The average Ashcroft score was calculated by averaging the individual field scores.

*Immunohistochemistry – For* C/EBPδ immunohistochemistry, 5-μm sections of paraformaldehyde fixed and paraffin embedded human control and IPF lungs were first deparaffinized and rehydrated. Subsequently, endogenous peroxidase activity was quenched using 1% H2O2 in methanol, slides were blocked with antibody diluent for 30 minutes and incubated with a rabbit polyclonal

antibody against C/EBPδ (GWB-MM818H; Genway Biotech, San Diego, CA, USA) at 1μg/mL in antibody diluent at 4 °C overnight. Next, slides were incubated with Brightvision poly-HRP anti rabbit IgG (DPVM-55HRP; Immunologic, the Netherlands) for 30 min at room temperature and stained with 3-3′ diaminobenzidine dihydrochloride (BS04-999; Immunologic, the Netherlands). Hematoxylin was applied as a counterstain.

*Quantitative real-time PCR* - Total RNA was isolated from lung homogenates using Tripure (Roche, Almere, Netherlands) according the manufacturer’s recommendations. All RNA samples were quantified by spectrophotometry and stored at -80°C until further analysis. mRNA was DNAse treated (#M6101; Promega, the Netherlands) after which cDNA was prepared according routine procedures. Gene expression analysis was performed using a Roche LightCycler480 thermocycler with SensiFAST Real-time PCR kit (#CSA-01190; Bioline, London, UK) using the gene specific primers listed in table 2. For c/ebpδ the specific Quantitect primer assay was used (Qiagen, Hilden, Germany). The results were normalized to tbp (for mouse samples), Ubiquitin (ubc) (for human samples) expression levels or to the general macrophage marker f4/80 for macrophage differentiation markers. The average Ct values for ubc and tbp were similar between groups.

*Statistics* - Statistical analyses were conducted using GraphPad Prism (GraphPad software, San Diego, CA, USA). Data are expressed as box and whiskers showing all points. Comparisons between two conditions are analyzed using two tailed unpaired t-tests when the data where normally distributed, otherwise Mann-Whitney analysis was performed. P values of less than 0.05 were considered significant.

## 3 Results

### 3.1 C/EBPδ expression in idiopathic pulmonary fibrosis

**Figure 1.**
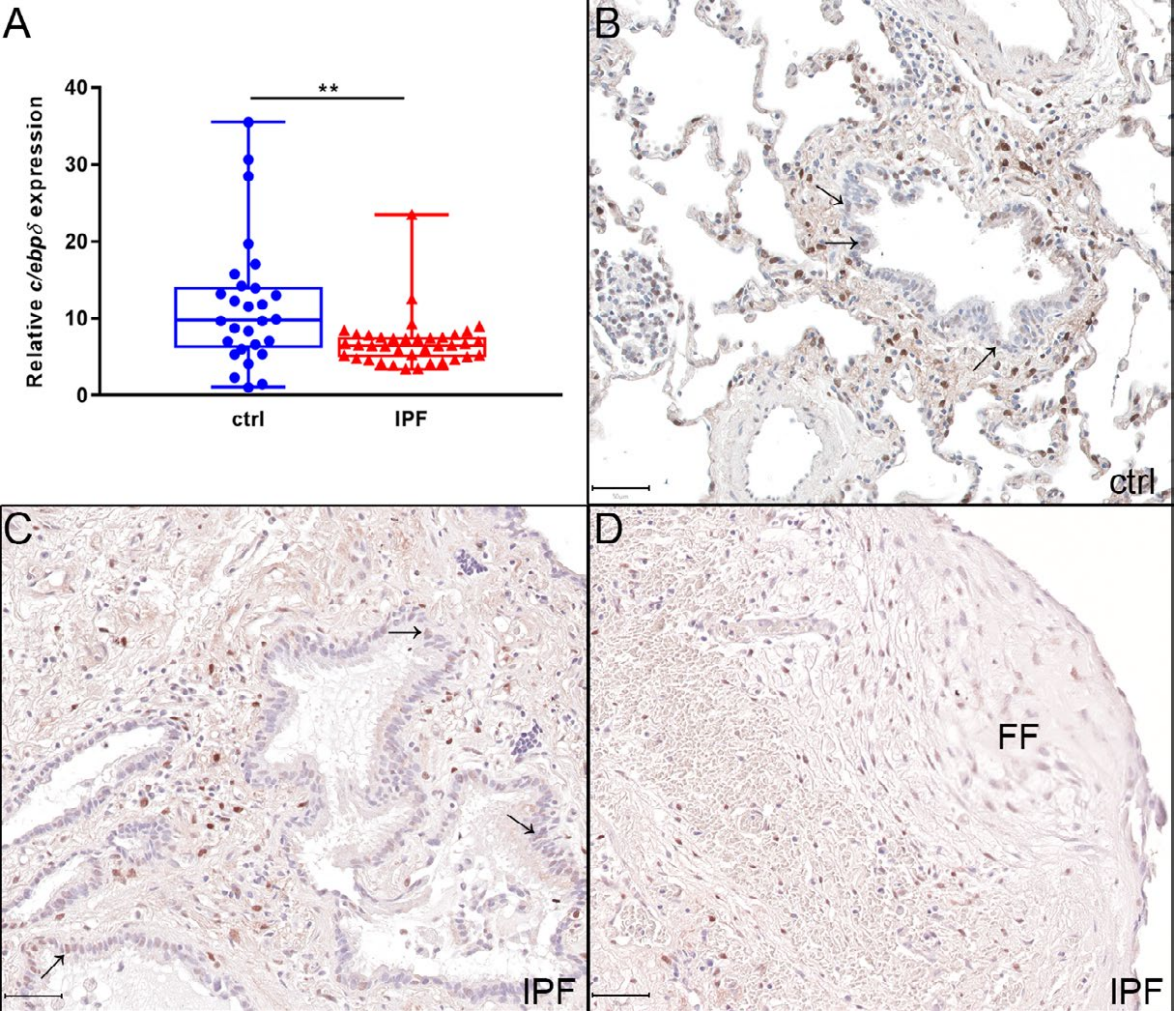
C/EBPδ expression is decreased in idiopathic pulmonary fibrosis (IPF) lung as compared to control (ctrl). (A) Quantitative PCR analysis of relative mRNA levels of C/EBPδ in whole lung extracts of control (●) and IPF (▲) tissue. Expression levels are relative to that of UBC. Data are represented as box and whiskers showing all points. n = 28-38. ** p<0.01. (B-D) Immunohistochemical staining of C/EBPδ protein expression in control (B) and IPF (C-D) lungs. Staining was observed in cells within connective tissue surrounding the small airways and bronchiolar epithelial cells (Arrow (→)). Very low staining was observed in fibroblast foci (FF). Scale bar: 50 μM.

To determine whether the expression of C/EBPδ is altered during pulmonary fibrosis, we assessed *c/ebpδ* expression levels in IPF patients and matched controls. As shown in Figure 1A, C/EBPδ expression in IPF patients was decreased by 1.6-fold as compared to control patients. In order to identify the cell type expressing C/EBPδ, we performed immunohistochemistry on control and IPF lung. As shown in Figure 1, C/EBPδ was strongly expressed in the connective tissue surrounding the small airways, like macrophages, fibroblasts and/or lymphocytes in control lung (Figure 1B). Additionally, C/EBPδ was weakly expressed in epithelial structures of the small airways, but no expression was observed in alveolar epithelial cells. In IPF lung (Figure 1C), a similar staining pattern was found with a strong expression of C/EBPδ in cells within the connective tissue surrounding the small airways, like macrophages, fibroblasts and lymphocytes, and a weak expression in bronchial/bronchiolar epithelium. Interestingly, within fibroblast foci ( FF; Figure 1D), C/EBPδ expression was very weak, also in the aforementioned cell types that are positive in connective tissue surrounding the small airways.

### 3.2 C/EBPδ does not affect fibrosis progression in bleomycin-induced pulmonary fibrosis

**Figure 2.**
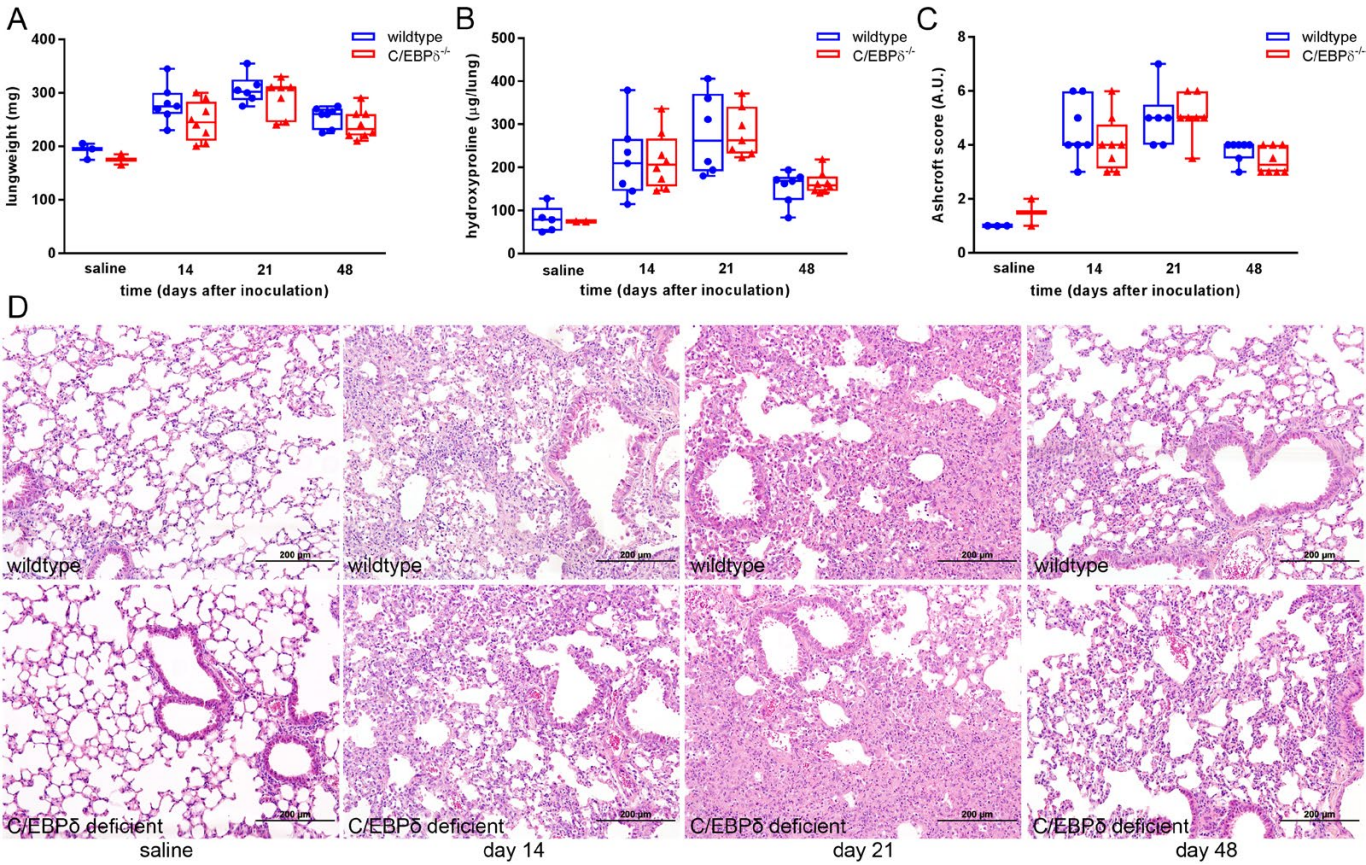
C/EBPδ deficiency does not affect bleomycin- induced pulmonary fibrosis. (A) lung weight (mg) of wildtype (●) and C/EBPδ deficient (▲) mice upon bleomycin instillation. (B) Collagen expression as measured by hydroxyproline levels in the right lung of wildtype (●) and C/EBPδ deficient (▲) mice upon bleomycin instillation. (C) Quantification of pulmonary fibrosis using the Ashcroft score in wildtype (●) and C/EBPδ deficient (▲) mice upon bleomycin instillation. (D) Representative H&E-stained lung tissue sections obtained after 14, 21 and 48 days of saline (left) or bleomycin instillation in wildtype (upper row) and C/EBPδ (lower row) mice. Data are represented as box and whiskers showing all points. n = 2-3 for saline and 6-8 for bleomycin.

**Figure 3.**
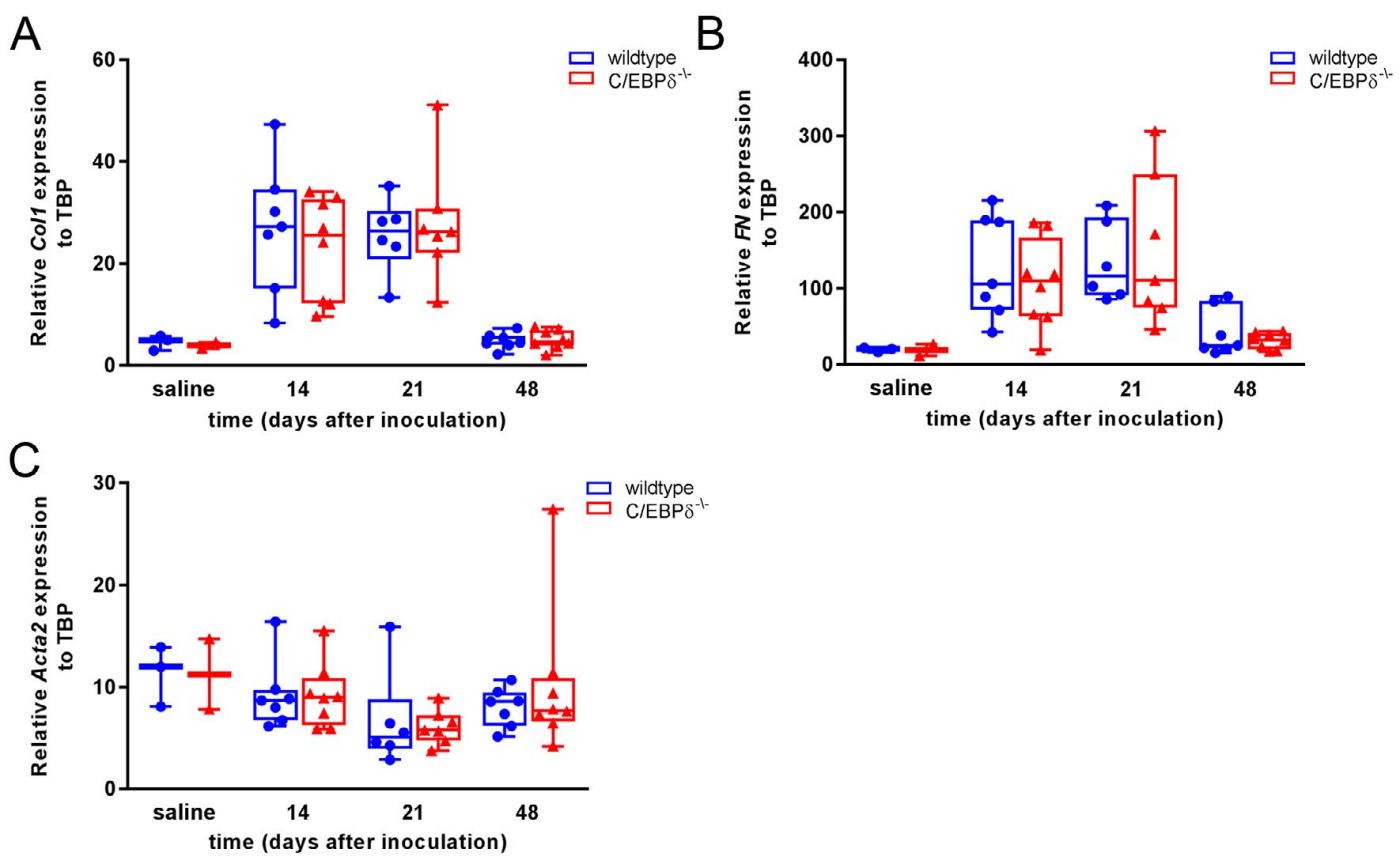
C/EBPδ deficiency does not affect fibrotic gene expression in bleomycin-induced pulmonary fibrosis. Quantitative PCR analysis of relative mRNA levels of collagen 1 (Col1) (A), fibronectin (FN) (B) and alpha-smooth muscle actin (ACTA2) (C) in whole lung extracts of wildtype (●) and C/ EBPδ deficient (▲) mice upon bleomycin instillation. Expression levels are relative to that of TATA-box binding protein (TBP). Data are represented as box and whiskers showing all points. n = 2-3 for saline and 6-8 for bleomycin.

In order to assess the role of C/EBPδ during the progression of pulmonary fibrosis, wildtype and C/EBPδ deficient mice were subjected to bleomycin- induced lung fibrosis for 14, 21 and 48 days. As shown in Figure 2, bleomycin induced extensive patchy areas of fibrosis culminating in severe pulmonary fibrosis at day 21 with some degree of resolution at day 48 post bleomycin inoculation. Importantly however, fibrosis progressed similarly in wildtype and C/EBPδ deficient mice as evident from similar increases in lung weight (Figure 2A), hydroxyproline levels representing collagen deposition (Figure 2B ) and histological injury (Figure 2C for Ashcroft scores and Figure 2D for representative H&E stainings). To substantiate these findings, we next assessed collagen 1 (Figure 3A), fibronectin (Figure 3B) and α-smooth muscle actin (Figure 3C) mRNA expression levels. As shown in Figure 3, collagen 1 and fibronectin expression increased up to 21 days post bleomycin inoculation but again no differences were observed between wildtype and C/EBPδ deficient mice. α-Smooth muscle actin, a marker for myofibroblast differentiation, expression was not increased during the progression of pulmonary fibrosis and its expression was similar in wildtype and C/EBPδ-/ - mice. Overall, these data show that C/ EBPδ does not play an important role in the progression of bleomycin-induced pulmonary fibrosis.

### 3.3 C/EBPδ does not affect macrophage differen-tiation or migration during bleomycin-induced lung fibrosis

**Figure 4.**
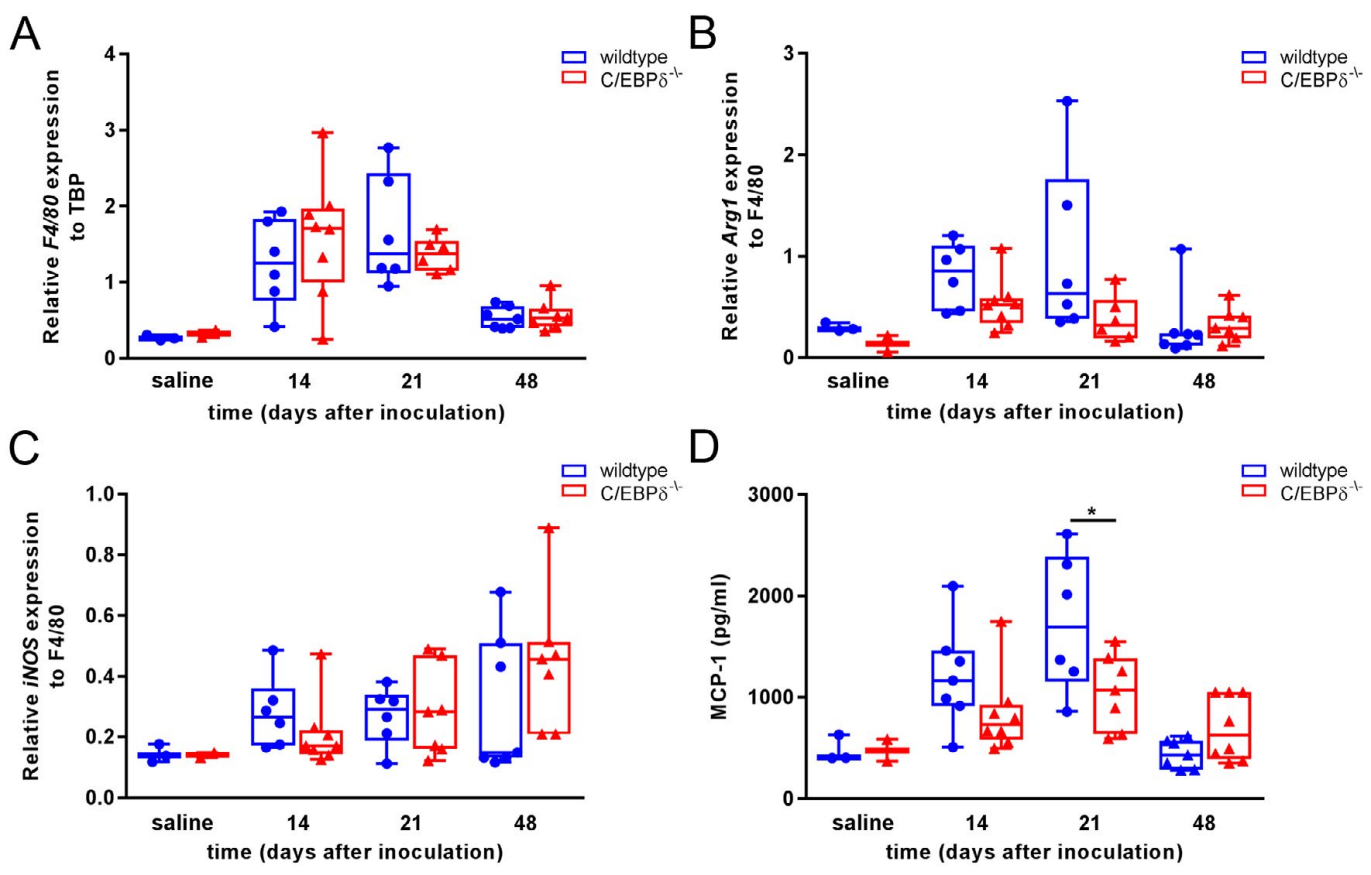
C/EBPδ deficiency does not affect macrophage migration or polarization in bleomycin-induced pulmonary fibrosis. Quantitative PCR analysis of relative mRNA levels of adhesion G protein-coupled receptor E1 (F4/80) (A), arginase (Arg1) (B) and inducible nitric oxide sythase 2 (iNOS) (C) in whole lung extracts of wildtype (●) and C/EBPδ deficient (▲) mice upon bleomycin instillation. F4/80 levels are relative to that of TATA-box binding protein (TBP). Arg1 and iNOS levels are relative to that of F4/80. (D) Monocyte chemotactic protein 1 (MCP-1) protein expression levels in whole lung extracts of wildtype (●) and C/EBPδ deficient (▲) mice upon bleomycin instillation. Data are represented as box and whiskers showing all points. n = 2-3 for saline and 6-8 for bleomycin. * p<0.05.

Macrophage recruitment in response to inflammatory mediators produced by injured epithelial cells is a key process in fibrosis Macrophage recruitment in response to inflam-matory mediators pro -duced by injured epithelial cells is a key process in fibrosis Macrophage recruitment by injured epithelial cells with subsequent macrophage differentiation is a hallmark of pulmonary fibrosis, whereas C/EBPδ has been implicated in both these processes. Consequently, we next assessed macrophage recruitment and/or differentiation during bleomycin-induced pulmonary fibrosis in both wildtype and C/EBPδ deficient mice. As shown in Figure 4A, macrophage numbers increased during fibrosis progression with a peak at day 21 post bleomycin inoculation. Again however, no differences were observed between wildtype and C/EBPδ deficient mice. In addition, macrophage differentiation as determined by iNOS (M1 macrophage marker) and Arg1 (M2 macrophage marker) expression levels was not significantly different between wildtype and C/EBPδ deficient mice (Figure 4B and C). Monocyte chemoattractant protein (MCP-1/CCL2) is an important cytokine for monocyte/macrophage recruitment and primarily produced by these cells. As shown in Figure 4D, MCP-1 expression was increased during fibrosis progression. Notably, MCP-1 were slightly decreased in C/ EBPδ deficient mice at day 21 after bleomycin instillation. Overall, C/EBPδ does not seem to modify macrophage influx and/or differentiation during experimental pulmonary fibrosis.

**Figure 5.**
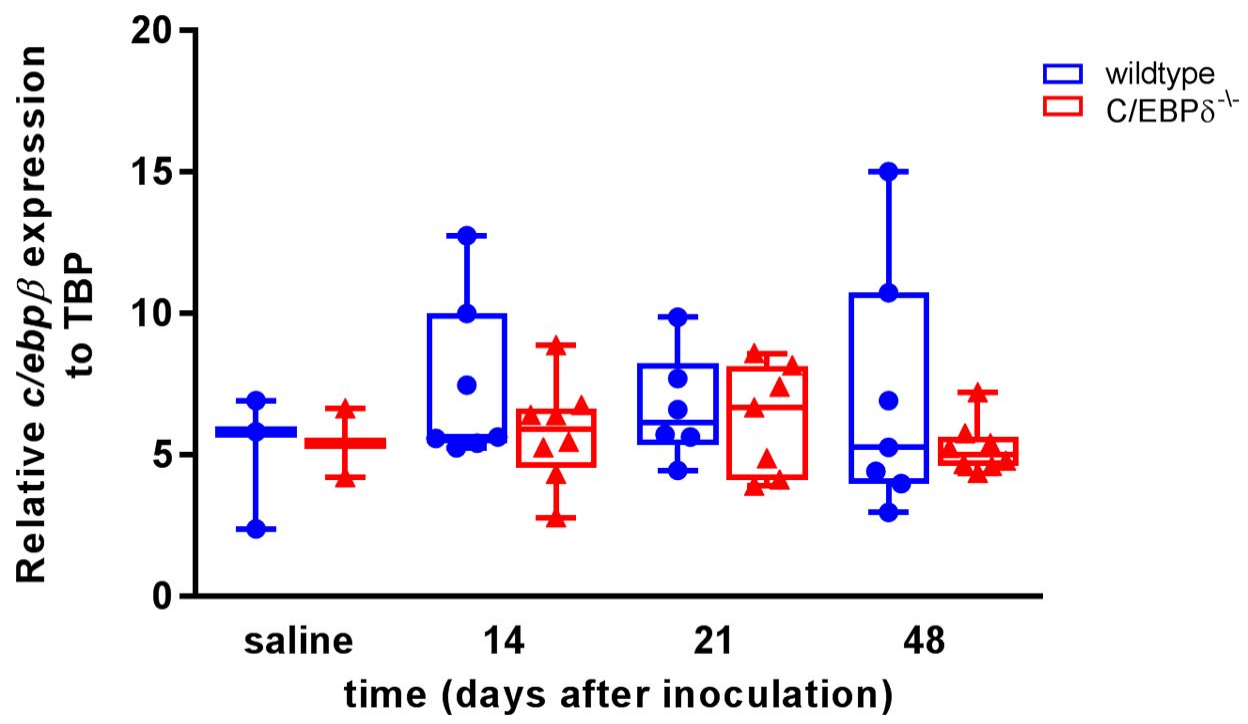
C/EBPβ expression during bleomycin-induced pulmonary fibrosis in wildtype and C/EBPδ deficient mice. Quantitative PCR analysis of relative mRNA levels of C/EBPβ in whole lung extracts of wildtype (●) and C/EBPδ deficient (▲) mice upon bleomycin instillation. Data are represented as box and whiskers showing all points. n = 2-3 for saline and 6-8 for bleomycin.

### 3.4 C/EBPδ and C/EBPβ may be redundant during pulmonary ibrosis.

Previous reports have shown that C/EBPα, -β and – 𝛅 are redundant for lipopolysaccharide-induced cytokine production [26,27] and the lack of effect of C/EBPδ deficiency on pulmonary fibrosis may thus result from some kind of redundancy between family members. Using in silico analysis of publically available GEOdatasets of IPF mRNA expression data, we identified C/EBPβ to be the most likely candidate for the compensatory loss of C/EBPδ. Indeed, C/EBPδ and C/EBPβ were the most abundant C/EBP family members, whereas C/EBPα (16x lower) and C/EBPε (250x lower) were less abundantly expressed in IPF lung (data not shown). Subsequent, quantitative PCR analysis of C/EBPβ expression revealed that C/EBPβ is indeed abundantly expressed in both wildtype and C/EBPδ deficient mice (Figure 5) suggesting that C/EBPβ might be a likely candidate to compensate for the loss of C/EBPδ during bleomycin-induced pulmonary fibrosis.

## 4 Discussion

C/EBPδ has been suggested to be a key regulator of cellular processes involved in pulmonary fibrosis and C/EBPδ may thus be an interesting target to pursue as a potential novel treatment modality. In the current manuscript, we however show that C/EBPδ deficiency has no effect on bleomycin-induced pulmonary fibrosis. We thus question the importance of C/EBPδ in pulmonary fibrosis and C/EBPδ may not be the most promising target to pursue.

Interestingly, we show that C/EBPδ expression was decreased in the lungs of IPF patients as compared to control patients. This is in line with a recent integrated genomic analysis in which C/EBPδ was identified as a transcription factor that was downregulated in IPF and was associated significantly with genes/pathways involved in fibrosis [10]. Although this association is noteworthy, the most important finding of our study is that the progression of fibrosis is similar in wildtype and C/EBPδ deficient mice and therefore C/ EBPδ does not seem to play a role in pulmonary fibrogenesis. Indeed, lung injury and ECM production increased over time, peaking at 21 days, to a similar extent in both wildtype and C/ EBPδ deficient mice. As already mentioned, C/EBPδ is part of a family of transcription factors which all interact with similar DNA binding sites. Consequently, the deficiency of a single family member may be compensated by other family members. Indeed, lipopolysaccharide-induced inflammatory cytokine production is subject to redundancy of C/EBPα, -β, and –δ [26,27]. The lack of effect of C/EBPδ deficiency on pulmonary fibrosis may thus result from some kind of redundancy between family members.

As opposed to the results of the current manuscript, we previously showed that C/EBPδ inhibits renal fibrosis. Indeed, C/EBPδ deficiency resulted in a more profound fibrotic response as evident from increased collagen deposition, tubular injury and transforming growth factor-β expression [28]. Apparently, the involvement of C/EBPδ in the progression of fibrosis is tissue specific and C/EBPδ does not seem a general anti-fibrotic mediator. A potential explanation for these discrepant results may be that redundancy occurs in a tissue specific manner due to differential expression levels of individual family members.

Macrophages are key regulators in the progression of pulmonary fibrosis [15-19]. Indeed, abundant numbers of activated macrophages are present in areas of fibrotic lung in both human IPF patients and bleomycin-challenged mice [29]. This activated M2-like macrophage population produces transforming growth factor beta, PDGF and CCL18, potent pro-fibrotic cytokines that induce fibroblast proliferation and differentiation into collagen-secreting myofibroblasts leading to ECM deposition and fibrosis [19]. Interestingly, C/EBPδ has been shown to promote the differentiation of bone-marrow- derived macrophages to the classical M1 type, while c/ebpδ silencing promotes the alternatively activated M2-type [30]. Here we show however that C/EBPδ does not significantly affect macrophage differentiation in the setting of pulmonary fibrosis. iNOS (M1 marker) expression levels are similar in wildtype and C/EBPδ deficient mice, whereas Arg1 (M2 marker) expression levels may be slightly decreased in C/ EBPδ deficient mice but this decrease is not statistically significant. Even if C/EBPδ would slightly modify macrophage differentiation, most importantly this does not modify the progression of pulmonary fibrosis.

Overall, we show that C/EBPδ does not affect experimental pulmonary fibrosis and we suggest that C/EBPδ may not be the eagerly awaited target to combat pulmonary fibrosis.

## References

[1] King TE Jr, Pardo A, Selman M (2011). Idiopathic Pulmonary Fibrosis.. Lancet.

[2] Gross TJ, Hunninghake GW (2001). Idiopathic Pulmonary Fibrosis.. N Engl J Med.

[3] du Bois RM (2010). Strategies for treating idiopathic pulmonary
fibrosis.. Nat Rev Drug Discov.

[4] Richeldi L, du Bois RM, Raghu G (2014). Efficacy and safety of nintedanib in idiopathic pulmonary
fibrosis.. N Engl J Med.

[5] King TE Jr, Bradford WZ, Castro-Bernardini S (2014). A phase 3 trial of pirfenidone in patients with idiopathic
pulmonary fibrosis.. N Engl J Med.

[6] Wuyts WA, Antoniou KM, Borensztajn K (2014). Combination therapy: the future of management for idiopathic
pulmonary fibrosis?. Lancet Respir Med.

[7] Lekstrom-Himes J, Xanthopoulos KG (1998). Biological role of the CCAAT/enhancer-binding protein family of
transcription factors.. J Biol Chem.

[8] Juan TS, Wilson DR, Wilde MD, Darlington GJ (1993). Participation of the transcription factor C/EBP delta in the
acute-phase regulation of the human gene for complement component
C3.. Proc Natl Acad Sci USA.

[9] Balamurugan K, Sterneck E (2013). The many faces of C/EBPδ and their relevance for
inflammation and cancer.. Int J Biol Sci.

[10] Gangwar I, Kumar Sharma N, Panzade G (2017). Detecting the Molecular System Signatures of Idiopathic Pulmonary
Fibrosis through Integrated Genomic Analysis.. Sci Rep.

[11] Duitman J, Hoogendijk AJ, Groot AP (2012). CCAAT-enhancer binding protein delta C/EBPdelta protects against
Klebsiella pneumoniae-induced pulmonary infection: potential role for
macrophage migration.. J Infect Dis.

[12] Chang LH, Huang HS, Wu PT (2012). Role of macrophage CCAAT/enhancer binding protein delta in the
pathogenesis of rheumatoid arthritis in collagen-induced arthritic
mice.. Plos ONE.

[13] Yan C, Johnson PF, Tang H (2012). CCAAT/enhancer-binding protein delta is a critical mediator of
lipopolysaccharide-induced acute lung injury.. Am J Pathol.

[14] Banerjee S, Xie N, Cui H (2013). MicroRNA let-7c regulates macrophage
polarization.. J Immunol.

[15] O’Dwyer DN, Ashley SL, Moore BB (2016). Influences of innate immunity, autophagy, and fibroblast
activation in the pathogenesis of lung fibrosis.. Am J Physiol Lung Cell Mol Physiol.

[16] Song E, Ouyang N, Hörbelt M (2000). Influence of alternatively and classically activated macrophages
on fibrogenic activities of human fibroblasts.. Cell Immunol.

[17] Wynn T A (2008). Cellular and molecular mechanisms of fibrosis.. J Pathol.

[18] Lin C, Rezaee F, Waasdorp M (2015). Protease activated receptor-1 regulates macrophage-mediated
cellular senescence: a risk for idiopathic pulmonary
fibrosis.. Oncotarget.

[19] Wynn TA, Barron L (2010). Macrophages: master regulators of inflammation and
fibrosis.. Semin Liver Dis.

[20] Lekkerkerker AN, Aarbiou J, van Es T, Janssen RA (2012). Cellular players in lung fibrosis.. Curr Pharm Des.

[21] Bardou O, Menou A, François C (2016). Membrane-anchored Serine Protease Matriptase Is a Trigger of
Pulmonary Fibrogenesis.. Am J Respir Crit Care Med.

[22] Sterneck E, Paylor R, Jackson-Lewis V (1998). Selectively enhanced contextual fear conditioning in mice lacking
the transcriptional regulator CCAAT/enhancer binding protein
delta.. Proc Natl Acad Sci USA.

[23] Lin C, Duitman J, Daalhuisen J (2014). Targeting protease activated receptor-1 with P1pal-12 limits
bleomycin-induced pulmonary fibrosis.. Thorax.

[24] Lin C, von der Thüsen J, Isermann B (2016). High endogenous activated protein C levels attenuates
bleomycin-induced pulmonary fibrosis.. J Cell Mol Med.

[25] Ashcroft T, Simpson JM, Timbrell V (1988). Simple method of estimating severity of pulmonary fibrosis on a
numerical scale.. J Clin Pathol.

[26] Hu HM, Baer M, Williams SC (1998). Redundancy of C/EBP alpha, -beta, and -delta in supporting the
lipopolysaccharide-induced transcription of IL-6 and monocyte
chemoattractant protein-1.. J Immunol.

[27] Lu YC, Kim I, Lye E (2009). Differential role for c-Rel and C/ EBPbeta/delta in TLR-mediated
induction of proinflammatory cytokines.. J Immunol.

[28] Duitman J, Borensztajn KS, Pulskens WP (2014). CCAAT-enhancer binding protein delta (C/EBPδ) attenuates
tubular injury and tubulointerstitial fibrogenesis during chronic
obstructive nephropathy.. Lab Invest.

[29] Withana NP, Ma X, McGuire HM (2016). Non-invasive Imaging of Idiopathic Pulmonary Fibrosis Using
Cathepsin Protease Probes.. Sci Rep.

[30] Banerjee S, Xie N, Cui H (2013). MicroRNA let-7c regulates macrophage
polarization.. J Immunol.

